# Draft Genome Sequences of Two Carbapenemase-Producing Klebsiella pneumoniae Strains Isolated from Blood Cultures

**DOI:** 10.1128/MRA.01057-18

**Published:** 2018-08-30

**Authors:** Rémi Le Guern, Teddy Grandjean, Karine Faure, Marvin Bauduin, Eric Kipnis, Rodrigue Dessein

**Affiliations:** a Recherche Translationnelle: Relations Hôte Pathogènes, University of Lille, Lille, France; b Institut de Microbiologie, CHU Lille, Lille, France; c Unité de Maladies Infectieuses, CHU Lille, Lille, France; d Service de Réanimation Chirurgicale, CHU Lille, Lille, France; Georgia Institute of Technology

## Abstract

Carbapenemase-producing Klebsiella pneumoniae represents an emerging public health issue. Here, we present the draft whole-genome sequences of K. pneumoniae clinical strains KPL0.1 (OXA-48 carbapenemase) and KPL0.2 (NDM-1 carbapenemase). These genome sequences should help in investigating pathophysiological mechanisms of digestive colonization or infection with these highly resistant bacteria.

## Abstract (precis)

Carbapenemase-producing Klebsiella pneumoniae represents an emerging public health issue. Here, we present the draft whole-genome sequences of K. pneumoniae clinical strains KPL0.1 (OXA-48 carbapenemase) and KPL0.2 (NDM-1 carbapenemase).

## ANNOUNCEMENT

Carbapenemase-producing *Enterobacteriaceae* (CPE) are resistant to most beta-lactams, including last-line options such as carbapenems, and are an emerging public health issue (1). Klebsiella pneumoniae colonizes the human gastrointestinal tract and can persist in the hospital environment, and outbreaks of carbapenemase-producing K. pneumoniae are frequently described (2). In France, the most frequently identified carbapenemases are OXA-48 (86% of the CPE) and NDM (9% of the CPE) (3). Two strains of carbapenemase-producing K. pneumoniae were isolated in the Lille University Teaching Hospital (CHU Lille, France), KPL0.1 (producing OXA-48 carbapenemase) and KPL0.2 (producing NDM-1 carbapenemase). Both strains were isolated from blood cultures using the Virtuo automated system (bioMérieux, Marcy-l’Étoile, France) and identified by matrix-assisted laser desorption ionization–time of flight (MALDI-TOF) mass spectrometry (Bruker Daltonics, Inc., Billerica, MA). KPL0.1 was isolated from a 77-year-old patient following digestive surgery, and KPL0.2 came from an 84-year-old patient with a urinary tract infection.

The DNA of each isolate was extracted using a QIAamp DNA minikit (Qiagen, Hilden, Germany), and quality control was performed using a Qubit v3.0 fluorometer (Thermo Fisher Scientific, Waltham, MA). Libraries were prepared using the Nextera XT DNA Library prep kit (Illumina, San Diego, CA), followed by paired-end (2 × 150-bp) sequencing on a HiSeq platform (Illumina). Genome coverage was about 650× for each isolate. Paired reads were filtered, and Nextera adapters were removed using Trimmomatic on a Galaxy server using default settings (4). Processed reads were *de novo* assembled with Unicycler. Genomes were annotated using the Prokaryotic Genome Annotation Pipeline (PGAP) v4.5 (5).

The draft genome of KPL0.1 consists of 5,825,863 bp and has a mean G+C content of 56.70%. A total of 5,628 protein-coding genes were annotated, including 92 RNA-coding genes and 75 tRNAs, and the remaining genes were annotated as hypothetical proteins. KPL0.1 belongs to sequence type 307 (ST-307) and possesses the KL102 capsule locus (6). The following resistance genes were predicted using ResFinder v3.0 (7): five beta-lactam resistance genes (*bla*_OXA-48_, *bla*_SHV-28_, *bla*_TEM-1B_, *bla*_CTX-M-15_, *bla*_OXA-1_), four aminoglycoside resistance genes [*aac(3)-IIa*, *aph(3ʺ)-Ib*, *aph(6)-Id*, *aac(6ʹ)-Ib-cr*], five fluoroquinolone resistance genes [*qnrS1*, *qnrB1*, *aac(6’)-Ib-cr*, *oqxA*, *oqxB*], and individual antimicrobial resistance genes (fosfomycin, *fosA*; phenicol, *catB4*; sulfonamide, *sul2*; trimethoprim, *dfrA14*; tetracycline, *tetA*).

The draft genome of KPL0.2 consists of 5,741,089 bp with a mean G+C content of 56.94%. A total of 5,570 protein-coding genes were annotated, including 93 RNA-coding genes and 79 tRNAs, and the remaining genes were annotated as hypothetical proteins. KPL0.2 belongs to ST-147 and possesses the KL64 capsule locus. The following resistance genes were predicted: five beta-lactam resistance genes (*bla*_NDM-1_, *bla*_SHV-11_, *bla*_TEM-1B_, *bla*_CTX-M-15_, *bla*_OXA-1_), four aminoglycoside resistance genes [*aac(3)-IIa*, *strA*, *aph(6)-Id*, *aac(6ʹ)-Ib-cr*), four fluoroquinolone resistance genes [*qnrB1*, *aac(6ʹ)-Ib-cr*, *oqxA*, *oqxB*], and individual antimicrobial resistance genes (fosfomycin, *fosA*; phenicol, *catB4*, sulfonamide, *sul1*; trimethoprim, *dfrA1*; tetracycline, *tetA*).

ST-147 was identified as one of the three major STs of carbapenemase-producing K. pneumoniae in a worldwide study; ST-147 is usually recovered from India, Italy, and Greece (8). ST-307 is described as highly prevalent in Italy (9). Overall, these two strains are representative of the carbapenemase-producing K. pneumoniae isolates in Europe. Further studies are warranted to investigate the pathophysiological mechanisms of digestive colonization or infection with these highly resistant bacteria.

### Data availability.

These two draft whole-genome shotgun projects have been deposited at DDBJ/ENA/GenBank under the accession numbers QHMA00000000 (KPL0.1) and PYBH00000000 (KPL0.2). The versions described in this paper are QHMA01000000 (KPL0.1) and PYBH01000000 (KPL0.2). The SRA accession number is SRP155589.
